# Exercise Training Attenuates the Dysregulated Expression of Adipokines and Oxidative Stress in White Adipose Tissue

**DOI:** 10.1155/2017/9410954

**Published:** 2017-01-12

**Authors:** Takuya Sakurai, Junetsu Ogasawara, Ken Shirato, Tetsuya Izawa, Shuji Oh-ishi, Yoshinaga Ishibashi, Zsolt Radák, Hideki Ohno, Takako Kizaki

**Affiliations:** ^1^Department of Molecular Predictive Medicine and Sport Science, Kyorin University, School of Medicine, 6-20-2 Shinkawa, Mitaka, Tokyo, Japan; ^2^Department of Sports Biochemistry, Faculty of Health and Sport Science, Doshisha University, 1-3 Tatara Miyakodani, Kyotanabe, Kyoto, Japan; ^3^National Hospital Organization Ibarakihigashi National Hospital, The Center of Chest Diseases and Severe Motor and Intellectual Disabilities, Terunuma 825, Tokai-mura, Naka-gun, Ibaraki, Japan; ^4^Institute of Sport Science, University of Physical Education, Alkotas u. 44, TF, Budapest, Hungary; ^5^Social Medical Corporation, The Yamatokai Foundation, Nangai, Higashiyamato, Tokyo, Japan

## Abstract

Obesity-induced inflammatory changes in white adipose tissue (WAT), which caused dysregulated expression of inflammation-related adipokines involving tumor necrosis factor-*α* and monocyte chemoattractant protein-1, contribute to the development of insulin resistance. Moreover, current literature reports state that WAT generates reactive oxygen species (ROS), and the enhanced production of ROS in obese WAT has been closely associated with the dysregulated expression of adipokines in WAT. Therefore, the reduction in excess WAT and oxidative stress that results from obesity is thought to be one of the important strategies in preventing and improving lifestyle-related diseases. Exercise training (TR) not only brings about a decrease in WAT mass but also attenuates obesity-induced dysregulated expression of the adipokines in WAT. Furthermore, some reports indicate that TR affects the generation of oxidative stress in WAT. This review outlines the impact of TR on the expression of inflammation-related adipokines and oxidative stress in WAT.

## 1. Introduction

In recent years, obesity due to hypertrophy of adipocytes caused by a lack of physical exercise and high-calorie diets has greatly increased in the world and has become a serious social problem [1]. The world obesity population has rapidly increased for the past 40 years, and now 10.8% of male adults (266 million) and 14.9% of female adults (375 million) are obese. If the trend continues, 18% of male adults and 21% of female adults will be obese by 2025 [2]. It is widely known that obesity is a risk factor for the development of lifestyle-related diseases such as type 2 diabetes and hyperlipidemia and that obesity and type 2 diabetes are responsible for an increase in arteriosclerotic disease [3, 4]. The number of diabetic patients has increased worldwide to reach 422 million, which is a fourfold increase in approximately 35 years [5]. As mentioned above, the worldwide increase in the number of obese people and in lifestyle-related disease patients is a serious problem, and this is true from the aspect of personal suffering as well as from the economical aspect of medical costs. As obesity increases, medical costs related to it logically rise. For instance, medical costs related to obesity reached 147 billion dollars in the US in 2008, and annual medical costs for obese people are on average 1,429 dollars higher than costs for people whose weight is standard, indicating a need to establish effective strategies for the prevention and improvement of obesity and lifestyle-related diseases [6]. Accordingly, solving the mechanism of diabetic development and establishing strategies for the prevention and improvement of obesity and type 2 diabetes are urgent tasks.

**Figure 1:**
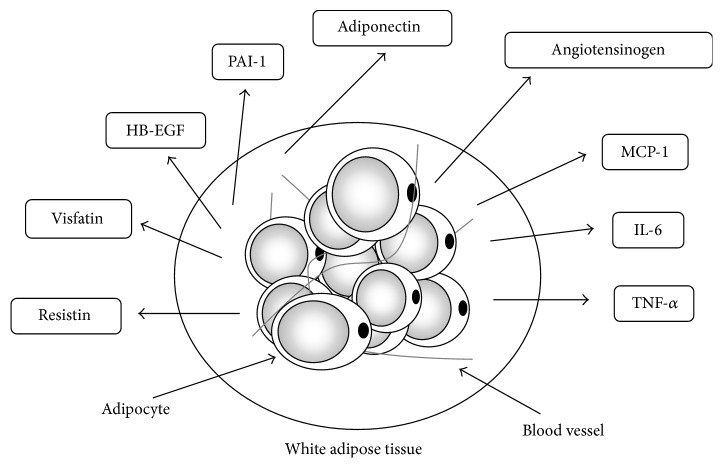
Various adipokines are secreted by white adipose tissue. White adipose tissue (WAT) secretes various humoral factors called adipokines: TNF-*α*, tumor necrosis factor-*α*; MCP-1, monocyte chemoattractant protein-1; IL-6, interleukin-6; HB-EGF, heparin-binding epidermal growth factor-like growth factor; and, PAI-1, plasminogen activator inhibitor-1. Adipokines are actively involved in metabolic reactions.

While white adipose tissue (WAT) was previously thought to be merely a matter of energy storage, recent examination at the molecular level has shown that adipocyte secretes various biologically active substances collectively known as “adipokines” (Figure 1) and that dysregulation of the expression of adipokines due to obesity is closely associated with the development of insulin resistance, which is a clinical condition of type 2 diabetes [7–9]. Therefore, WAT is thought to be one of the tissues that cause lifestyle-related diseases, and this has attached importance to both decreasing WAT mass and improving the disordered secretion of adipokines as strategies toward the prevention and improvement of lifestyle-related disease. It has become clear that oxidative stress is deeply involved in the dysfunction that includes the expression disorder of adipokines in WAT due to obesity [10–13]. On the other hand, exercise training (TR) not only decreases WAT mass but also affects the expression of various adipokines in WAT [14, 15]. Moreover, TR is now reported to affect the oxidative stress in WAT. This review outlines the mechanisms for the dysregulated expression of adipokines due to increases in oxidative stress in WAT and also reviews the effect of TR, which is a useful strategy for the prevention and improvement of obesity and type 2 diabetes.

## 2. Inflammatory Changes in WAT and Adipokines

**Figure 2:**
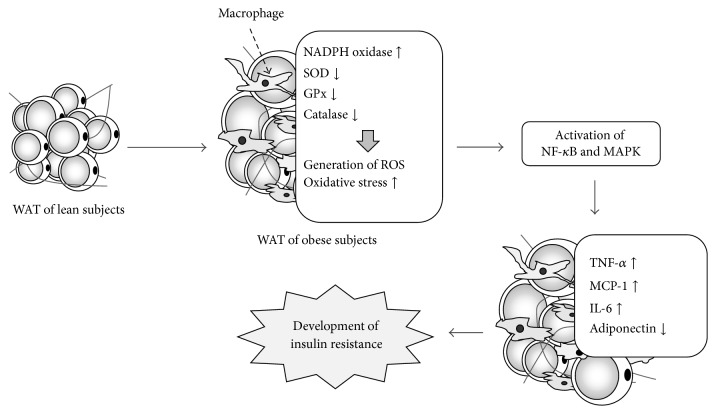
Model of the generation of oxidative stress and the development of chronic inflammation in obese WAT. Adipocytes begin to grow as a result of factors such as excess energy intake and lack of exercise, and macrophages infiltrate into WAT. Moreover, the expressions of NADPH oxidase subunits increase and those of antioxidants, such as superoxide dismutase (SOD), glutathione peroxidase (GPx), and catalase, decrease. As a result, reactive oxygen species (ROS) are produced in excess. Oxidative stress in obese WAT relates to dysregulated expression of inflammation-related adipokines via the activation of nuclear factor-*κ*B (NF-*κ*B) and mitogen-activated protein kinase (MAPK). The dysregulated expression of adipokines induces inflammation of WAT, contributing to the development of insulin resistance.

Obesity is a major cause of insulin resistance which is one of the type 2 diabetic clinical conditions. Recent studies have defined the role that chronic and mild systemic inflammation plays in the development of diabetic conditions. In fact, a rise in inflammatory markers such as C-reactive protein (CRP), haptoglobin, and fibrinogen in the blood is recognized in diabetic patients, and increases in interleukin- (IL-) 1*β*, IL-6, and CRP in the blood are predictive factors for type 2 diabetes [16–20]. Moreover, the blood concentration of macrophage migration inhibitory factor (MIF), which is a humoral factor that controls the migration of macrophage, in type 2 diabetic patients has been reported to be higher than that of healthy persons [21]. Furthermore, the blood levels of monocyte chemoattractant protein-1 (MCP-1), which is identified as a monocyte chemotactic factor, also rise in obese individuals [22]. On the other hand, a great deal of evidence shows that hypertrophic WAT itself shows inflammatory change [7–9]. When WAT is enlarged due to surplus energy intake or lack of physical exercise, an infiltration of macrophage, neutrophils, and T-cells is observed in WAT. In WAT infiltrated by macrophages, the production of proinflammatory adipokines, such as tumor necrosis factor-*α* (TNF-*α*) and MCP-1, is increased and the production of anti-inflammatory adiponectin is decreased, and then a chronic inflammatory condition develops (Figure 2). Increases in proinflammatory adipokines act not only on adipocytes but also on paracrine, skeletal muscle, and liver, bringing about insulin resistance in those organs [23–25].

## 3. Representative Adipokines and Their Functions 

### 3.1. TNF-*α*

There are a number of reports that focus on typical inflammatory adipokine TNF-*α* and its involvement in insulin resistance and other actions since the expression of TNF-*α* was found to be upregulated in the WAT of obese model animals [26, 27]. The expression of TNF-*α* increases in the WAT of obese animals as well as that of human WAT due to obesity, and the expression level of TNF-*α* in WAT shows a significant positive correlation with body mass index (BMI) and blood insulin concentration [28–30]. TNF-*α* attenuates insulin signaling via the insulin receptor substrate 1- (IRS-1-) mediated inhibition of insulin receptor tyrosine kinase activity in areas such as skeletal muscle, and it reduces the expression of glucose transporter (GLUT) 4 and adiponectin in adipocytes [31–34]. Therefore, increased TNF-*α* causes the development of insulin resistance in skeletal muscle and WAT. On the other hand, while a TNF-*α* knockout mouse showed the same level of obesity as a control mouse by means of a high fat diet (HFD) feeding, insulin resistance was not shown, and the GLUT4 protein contents in skeletal muscle increased [35]. Furthermore, tyrosine phosphorylation of an insulin receptor by insulin stimuli in visceral WAT and skeletal muscle was accelerated. Also, insulin resistance is shown to be weak in obese TNF-*α* receptor knockout mice [36]. Nevertheless, different reports coexist concerning the role of TNF-*α* in the cause of insulin resistance at the molecular level, which arises from actual cases where TNF-*α* in the blood was blocked. When glucose metabolism has improved, however, some studies have failed to demonstrate the beneficial effects on glucose metabolism caused by the blockage of TNF-*α* in the blood [37–42]. Therefore, further prospective studies are required.

### 3.2. MCP-1

Increases in the genetic manifestations of MCP-1 have been found in the WAT of obese mice [43, 44]. Also, a positive correlation has been established with the degree of obesity and the blood concentration of MCP-1, which induces the infiltration of macrophage into WAT via C-C chemokine receptor-2 (CCR2) and also plays a role in inflammatory changes [22, 45]. A mouse genetically modified for an enhanced expression of MCP-1 in adipocytes showed no differences in body mass, WAT mass, or the size of adipocytes compared with a control mouse, but the infiltration of macrophage into WAT was increased by comparison and an enhanced expression of TNF-*α* in WAT was observed [43]. Therefore, the infiltration of macrophage into WAT due to an increased MCP-1 signal was suggested to cause further production of inflammatory adipokines such as TNF-*α*. As a result of that study, the inflammatory response in WAT is thought to be an amplifier of these processes. Moreover, tyrosine phosphorylation of IRS-1 and phosphorylation of Akt, which are essential intermediate factors in insulin signaling, was reduced in the WAT of mice that were genetically modified to express only MCP-1 excessively in adipocytes [43]. In another study, when a MCP-1 knockout mouse was fed a HFD to the point of obesity, the infiltration of macrophage into WAT was controlled and insulin sensitively was increased in a more sensitive manner compared with that in a control mouse [44]. Another study found that, in CCR2 knockout mouse, infiltration of macrophage in WAT due to HFD feeding-induced obesity was reduced and insulin sensitively was enhanced by comparison with wild type mice [45]. CCR2 manifests itself in monocytes and macrophages as well as in adipocytes and skeletal muscle cells, and MCP-1 is suggested to directly act on those cells to attenuate the insulin signal [43].

### 3.3. Adiponectin

Adiponectin increases fatty acid oxidation and glucose uptake in skeletal muscle, and in the liver it exerts the inhibitory effect of gluconeogenesis [46, 47]. Adiponectin also inhibits the expression and secretion of TNF-*α* in macrophage and increases the production of anti-inflammatory cytokines, such as IL-10, while controlling the attachment of monocytes to vascular endothelial cells and the transformation of macrophage into foam cells [48, 49]. Thus, adiponectin is thought to have anti-inflammatory effects. In accordance with that function, the expression of gene for adiponectin is reduced in the WAT of genetically obese mice and obese humans, and both obese individuals and diabetic patients have a lower blood concentration of adiponectin compared with healthy individuals [50, 51]. Additionally, there is a negative correlation between the blood concentration of adiponectin and the degree of insulin resistance [52]. When a physiological concentration of adiponectin is administered to KKAy mice (model mice for type 2 diabetes), insulin resistance and hyperlipidemia are improved. On the other hand, adiponectin or its receptor (AdipoR) knockout mice has exhibited insulin resistance [46, 47]. These finding suggests that a decrease in adiponectin expression in WAT is closely associated with the cause of insulin resistance and the onset of type 2 diabetes. Also, adiponectin is suggested to be involved in mitochondrial biogenesis since a knockout mouse of AdipoR in skeletal muscle showed a reduced mitochondrial content, reduced type I muscle fibers, and decreased capacity for exercise [53]. A recent report stated that the oral administration of AdipoR agonist (AdipoRON) improved insulin resistance and glucose tolerance in a diabetic mouse, and clinical applications are expected [54, 55].

### 3.4. Leptin

Leptin is a hormone that is secreted from adipocytes, and leptin receptors (ob-R) that lay on the hypothalamus produce a strong suppression of feeding, a rise in energy consumption, and improvements in glycometabolism [56–58]. Although leptin gene expression is enhanced in the WAT of obese individuals and the blood concentration increases in obese individuals as well as in obese animals, the effects of a suppression of feeding and a rise in energy consumption are hampered by the appearance of a dysfunction of leptin that is referred to as “leptin resistance” [57, 59–61]. Therefore, obesity is not improved efficiently even though leptin concentration in the blood is high. On the other hand, it is suggested that leptin is related to immune reactions. In fact, thymic atrophy and immunodeficiency have been observed in leptin-deficient (ob/ob) mice and also in ob-R-deficient (db/db) mice [62]. Moreover, leptin is known to have an inflammatory function. Leptin increases inflammatory cytokines such as TNF-*α* and stimulates macrophages in mice to induce the production of chemokines [63–65]. Recently, leptin was suggested to be involved in a phenotype of infiltrated macrophages in the WAT of obese and diabetic mice [66].

### 3.5. IL-6

IL-6 is produced in various types of cells, such as adipocytes, lymphocytes, and macrophages, and is involved in functions such as immune responses and inflammation [67–69]. Furthermore, IL-6 acts on liver to increase production of the inflammation biomarker CRP [70–73]. Therefore, IL-6 is suggested to be deeply involved in the onset of autoimmune disease. The aberrational production of IL-6 is thought to be a cause of articular rheumatism [74]. IL-6 may be involved in the cause of insulin resistance since its blood concentration is high in obese individuals and in type 2 diabetic patients, and expression of the IL-6 gene is increased in the subcutaneous WAT of individuals with insulin resistance [75–77]. When IL-6 is added to adipocytes, the tyrosine phosphorylation of IRS-1 that is stimulated by insulin is weakened and glucose uptake into cells is attenuated due to a decrease in GLUT4 [78].

## 4. Overproduction of ROS in WAT due to Obesity

Obesity has been associated with systemic oxidative stress, and clinical studies have shown that the onset of metabolic syndrome and type 2 diabetes is involved in systemic oxidative stress [11, 12, 79, 80]. For instance, highly sensitive CRP and other oxidative stress markers, such as oxidized low-density lipoprotein, are high in obese individuals, and positive correlations with these oxidative stress markers and BMI and percent body fat have been observed [81]. Moreover, in type 2 diabetic patients, lipids, protein, and DNA oxidation are recognized in the blood, and a positive correlation has been noted between the degree of oxidation and glycemic control [13]. On the other hand, increased oxidative stress has been described in the WAT of obese animals. Enlarged adipocytes are a significant source of ROS and excessively produced ROS is deeply involved in the dysfunction of WAT, which includes insulin resistance and inflammatory reactions (Figure 2) [82]. In KKAy mice, elevated lipid oxidation and the generation of H_2_O_2_ are observed in WAT but neither in liver nor in skeletal muscles, and increases in oxidative stress have also been observed in the WAT of HFD and ob/ob mice [82–84].

ROS is believed to have different sources in WAT. The first ROS source is mitochondria. A mitochondrion is the main energy-producing organ and is also responsible for the generation of oxidative stress. During the process of energy production in mitochondria by oxidative phosphorylation, a portion of the oxygen molecule creates a superoxide. Therefore, an influx of excess nutrients to adipocytes is thought to bring about increases in the mitochondrial substrate load, which results in enhanced production of ROS in mitochondria [11]. In fact, highly concentrated glucose and the exposure of free fatty acids (FFA) increase ROS generation in mitochondria in 3T3-L1 adipocytes [85–87]. Also, oxidative stress in WAT is elevated when mice become hyperglycemic [88]. Additionally, it is understandable that enhanced oxidative stress in WAT induces mitochondrial dysfunction, which causes further increases in oxidative stress in WAT [89]. Nevertheless, some reports argue that FFA added to adipocytes is transformed into acyl-CoA without significant mitochondrial oxidation and that oxidative phosphorylation or beta-oxidation does not elevate when glucose and palmitic acid are added to adipocytes [90, 91]. Therefore, elucidation of the role that mitochondria plays in increasing ROS generation in WAT will require further study.

The second ROS source is due to NAPDH oxidase, which consists of seven member proteins (NOX1–5, DUOX1, and DUOX2), and is a major source of ROS production in various cells [92, 93]. Nox produces superoxide when it receives an electron from the NADPH of the cytoplasm and transfers it to oxygen. Once it is produced, superoxide is further transformed into H_2_O_2_. NAPDH oxidase is a source of ROS in adipocytes, and NOX4 is particularly essential for ROS generation in adipocytes [94, 95]. In fact, ROS generation from the stimulus of glucose and palmitic acid is decreased in adipocytes where NOX4 expression is reduced [91]. Moreover, the fact that mitochondrion causes ROS and is a target for ROS production by NAPDH oxidase suggests that there is cross talk between mitochondria and NAPDH oxidase. NADPH oxidase has been associated with an elevation of ROS generation in obese WAT. The WAT of obese mice has demonstrated increased expressions of NADPH oxidase subunits and decreases in antioxidants such as superoxide dismutase (SOD) and glutathione peroxidase, which led to lipid peroxidation and an elevation in H_2_O_2_ production (Figure 2) [84].

## 5. Dysregulated Production of Adipokines in WAT due to Oxidative Stress

Obesity-induced increase in oxidative stress in WAT is suggested to be one of the causes of the dysregulated expression of inflammatory-related adipokines (Figure 2). The enhanced expression of genes for inflammatory adipokines such as TNF-*α* and the decrease in anti-inflammatory adiponectin gene expression are seen in the WAT of KKAy mice, which is accompanied by high levels of oxidative stress [84]. Also, when an oxidant is directly added to cultured 3T3-L1 adipocytes, the expressions of the IL-6 and MCP-1 genes increase and that of adiponectin decreases [96, 97]. On the contrary, antioxidant treatment for adipocytes, such as polyphenol addition, definitely attenuates the expression of genes for TNF-*α* and MCP-1 in adipocytes [98, 99]. Members of the mitogen-activated protein kinase (MAPK) family, such as extracellular signal-regulated kinase (ERK) and c-Jun N-terminal kinase (JNK) and transcription factor nuclear factor-*κ*B (NF-*κ*B), are activated by oxidative stress and significant mediators of oxidative stress-induced intracellular signal transduction, and they are thought to play a significant role in the dysregulated expression of adipokines due to oxidative stress (Figure 2) [100, 101]. Oxidative stress activates NF-*κ*B in various cells and controls the expressions of TNF-*α* and MCP-1 [101–103]. Adding *N*-acetyl-cysteine (NAC), which is a strong antioxidant, weakens the activation of NF-*κ*B, and, as a result, the enhancement of plasminogen activator inhibitor-1 expression by TNF-*α* is attenuated [104]. On the other hand, ERK's activation is thought to be involved in the expression of MCP-1 in adipocytes [105]. Moreover, since a large number of inflammatory cytokines accelerate ROS generation in macrophage, monocytes, and vascular endothelial cells, inflammation-related cross talk between infiltrated macrophage and adipocytes is expected to be amplified in obese WAT.

## 6. The Effect of TR on Preventing and Improving Obesity and Type 2 Diabetes and the Expression of Adipokines in WAT

It is widely accepted that physical exercise is effective in preventing and improving obesity and lifestyle-related diseases. For instance, Helmrich et al. [106] tracked 5,990 graduates of Pennsylvania University for 14 years and reported that every 500 kcal increase in the amount of exercise reduces the risk of the development of diabetes by 6%. Also, according to a follow-up survey of 21,271 US male medical doctors, the risk of developing diabetes fell in the group that experienced exercise to the point of sweating at least once a week [107]. Moreover, Manson et al. [108] followed the progress of 87,253 US female nurses for 8 years and found that the relative risk of developing diabetes, for the group that exercised to the point of sweating at least once a week, dropped to 0.84 compared with the group that exercised less than once a week.

A large number of examinations as to effect of TR on the expression of adipokines in WAT and the blood level of adipokines have been conducted for both animal experiments and human studies. However, every report did not agree due to differences in experimental subjects, exercise intensity, or exercise duration [14]. For example, the enhanced expression of the gene for TNF-*α* in the mesenteric visceral WAT of HFD feeding-induced obese mice is reported to have been inhibited by spontaneous running exercise in a rotating cage over a period of 6 weeks [109]. Furthermore, in a similar report combining a HFD with treadmill running, there was no difference in TNF-*α* gene expression in the visceral WAT of mice between a HFD group that continued TR for 6 weeks and a group with a HFD alone. After further 6 weeks, however, the increased expression of the gene for TNF-*α* due to the HFD feeding was attenuated by TR [110]. We have also reported that TNF-*α* protein content in the visceral WAT of rats was decreased by making them run on treadmills for 9 weeks [111, 112]. Nevertheless, when mice are subjected to the combination of a high-sucrose diet and TR that consists of spontaneous running, increases in TNF-*α* genes and in protein expression are routinely observed in mesenteric visceral WAT by comparison with groups that have only a high-sucrose diet [113]. Lira et al. [114] also observed an increased amount of TNF-*α* in the mesenteric visceral WAT of rats subjected to 8 weeks of treadmill TR. In examinations of obese people, Bruun et al. [115] have reported that when TR, such as walking five times a week, and dietary manipulation were adopted by highly obese male and female adults for 15 weeks, TNF-*α* gene expression in subcutaneous WAT decreased. Other reports, however, have found that TNF-*α* in human subcutaneous WAT was not changed due to TR [116, 117]. Although the above-mentioned discrepancy about the effects of TR on the TNF-*α* expression in WAT could not be resolved at this time, one of the possible reasons for the discrepancy may be site differences in WAT because TR tends to attenuate the TNF-*α* expressions in the epididymal and retroperitoneal WAT but to increase mesenteric WAT [111–114, 116, 117]. Moreover, TR appears to be insensitive to the TNF-*α* contents in the subcutaneous WAT. In our experiment, TR did not affect the TNF-*α* protein content in the subcutaneous WAT of the rats, although the TNF-*α* protein expressions in the epididymal and retroperitoneal WAT were decreased by TR [111]. Adipokines have been reported to exhibit fat depot-specific expression [118]. For example, the TNF-*α* level in the mesenteric WAT is suggested to be higher than those in the omental and subcutaneous WAT [118–120]. Therefore, TR may induce fat depot-specific differences in adipokine expression. Besides adipokines, the adaptation of basal autophagic activity following TR has been reported recently to exhibit fat depot-specific differences [121]. On the other hand, there have been indications that obesity-induced increases in TNF-*α* levels in WAT are not due to adipocytes but rather to increased expression by infiltrating macrophages, based on an experiment that cocultured adipocyte and macrophage cell lines [122]. As Harman-Boehm et al. [123] reported that CD68-positive macrophages are more highly abundant in omental than in subcutaneous WAT in lean and obese subjects, differences in the number of infiltrating macrophages in each WAT are speculated to reflect the effects of TR on TNF-*α* expression. Similar to TNF-*α*, conflicting results have been obtained with regard to MCP-1. Expression of the MCP-1 gene increases in the visceral WAT of a mouse fed HFD, and this increased expression is attenuated by spontaneous running for 6 weeks [109]. Other examinations that have combined HFD feeding and TR that involved running on a treadmill have shown that MCP-1 gene expression, which normally increases due to HFD feeding, was decreased [110]. We also observed that MCP-1 protein contents in rat visceral WAT were reduced by 9 weeks of TR that involved running on a treadmill [111]. However, expression of the gene for MCP-1 was not altered in either the subcutaneous or visceral WAT of a rat engaged in spontaneous running TR for 4 weeks, and the same was true for obese individuals engaged in aerobic exercise for 12 weeks [116, 124]. Meanwhile, increased expression of the gene for adiponectin was observed in the subcutaneous WAT of obese individuals who underwent a combination of dietary therapy and TR such as walking 5 times a week for 15 weeks or endurance training for 12 weeks [115, 116, 125] Nevertheless, when obese women engaged in TR for 12 weeks a decrease in body fat was observed, but no effect was seen on adiponectin gene expression in subcutaneous WAT [117]. Examinations using rats have shown that the expression of genes for adiponectin in visceral and subcutaneous adipocytes was upregulated by means of TR that involved 9 weeks of running on a treadmill [126]. However, Gollisch et al. [124] have indicated that short-term (4 weeks) TR by self-propelled running inhibits HFD-induced upregulation of the gene expression of adiponectin in the subcutaneous WAT of rats. Interestingly, in this study, no effect of TR on the expression of the gene for adiponectin in visceral WAT was observed. Secretion and mRNA expression of adiponectin positively correlated with the size of isolated adipocytes in humans and rats [126, 127]. Nevertheless, the adiponectin expression levels in the WAT of genetically obese mice and obese humans are reduced [50, 51]. Therefore, reduced adiponectin expression in vivo, rather than increases in adipocyte size, is speculated to be the result of inflammatory adipokines such as TNF-*α* [128]. In fact, the TR upregulated expression of the gene for adiponectin in the subcutaneous WAT has been reported in obese humans with downregulation of TNF-*α* gene expression [115]. In the experiment about the early phase of obesity (4 weeks) by Gollisch et al. [124], the subcutaneous WAT mass was increased and the adipocyte number per gram of WAT tended to increase, but the inflammatory state in WAT was weak. Therefore, the effect of WAT expansion may be related to the enhancement of the adiponectin expression in subcutaneous WAT and TR appears to be able to inhibit the enhancement of adiponectin expression because TR attenuates increases in subcutaneous WAT due to HFD feeding.

## 7. The Effect of TR on Oxidative Stress in WAT

**Figure 3:**
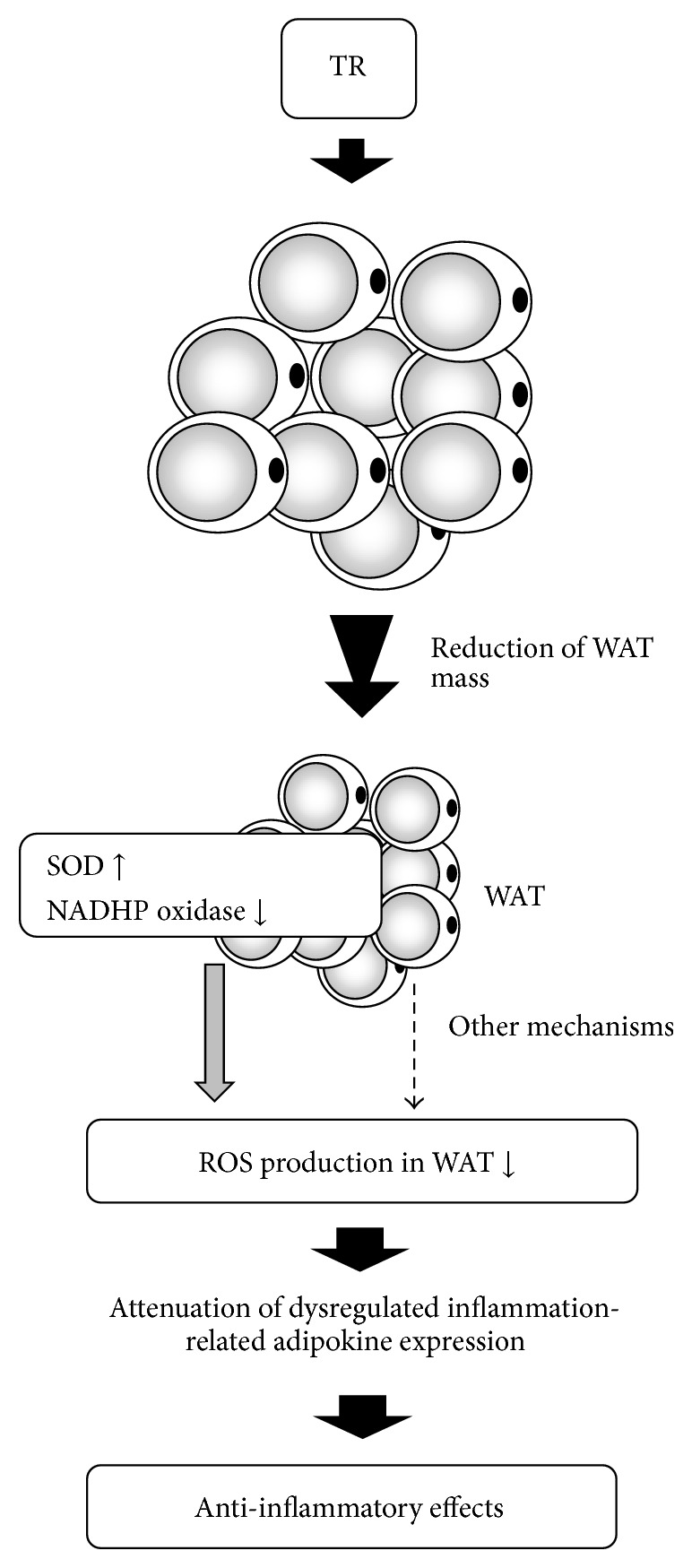
Schematic model for the antioxidative/anti-inflammatory effects of exercise training in WAT. Exercise training (TR) reduces WAT mass and attenuates an enhanced expression of NADPH oxidase and a decreased expression of Mn-SOD. Therefore, TR is thought to decrease the expression of inflammation-related adipokines via a reduction in oxidative stress in WAT.

The size of adipocytes greatly affects the expression of adipokines [127–130]. For example, in isolated human adipocytes, secretion levels of TNF-*α*, MCP-1, and IL-6 are positively correlated with cell size, and after correction for the cell surface area, there remains a significant difference between very large and small adipocytes for MCP-1 and IL-6 [129]. TR is well known to decrease WAT mass (size of adipocytes), and, therefore, it is understandable that the effect of TR on the dysregulated expression of adipokines in WAT largely depends on a decrease in adipocyte size. On the other hand, it seems that one of the unique effects of TR other than the reduction of WAT mass is to decrease oxidative stress in WAT (Figure 3). For example, we found that the values for lipid peroxidation in the epididymal and retroperitoneal WATs of rats engaged in TR are lower than in those of control rats and that TNF-*α* and MCP-1 content in WAT are less in the TR groups than in control groups [111]. Also, the phosphorylation of ERK, which is activated by oxidative stress and is involved in the expression of MCP-1, was attenuated by TR [105, 111]. Moreover, de Farias et al. [131] have also reported that the HFD feeding-induced increases in lipid peroxidation in mice WAT were significantly diminished by TR. The underlying mechanisms of the antioxidative effects of TR on WAT have been examined for an alternation of the NADPH oxidase and antioxidant enzymes. In our experiment mentioned above, the Mn-SOD content in WAT was increased due to TR, whereas NOX2 was reduced [111, 112]. Also, in examinations of obese mice, expression of the Mn-SOD gene was increased by TR and that of MCP-1 and NOX2 genes was decreased [132]. Additionally, when obese mice have been engaged in TR, enhanced enzymatic activity was observed for Mn-SOD and catalase [131]. Ferrara et al. [133] found that TR decreases oxidative stress with increases in Mn-SOD and catalase proteins in the WAT of old rats. However, at least one report has stated that even when conducted in combination with a HFD TR affects the gene expression of neither Mn-SOD nor NOX2, and, therefore, further examination is required [134].

There are many unclear points regarding whether the effect of TR is specific to the expressions of NADPH oxidase and antioxidant enzymes in WAT with the exception of decreases in WAT mass and whether the effects of hormesis by TR exist. As mentioned above, the expression of NADPH oxidase increases and that of antioxidant enzyme decreases in the WAT of obese mice, and, as a result, oxidative stress rises in WAT [84]. Moreover, as lipid droplets form, oxidative stress rises in 3T3-L1 adipocytes [84]. Since TR reduces WAT mass, it is possible that TR-induced decreases in the WAT mass cause changes in the expression of NADPH oxidase and antioxidant enzymes. On the other hand, many examinations have been conducted on the physical exercise-induced hormesis effect in skeletal muscles. Physical exercise increases oxidative stress in skeletal muscle, and then physical exercise inducing oxidative stress upregulates the expression of antioxidant enzymes in skeletal muscle; in other words, antioxidant adaptation against oxidative stress is caused by the physical exercise of skeletal muscle [135–139]. Since the amount of oxygen consumed in exercising skeletal muscle increases beyond the amount at rest, ROS production in the mitochondria increases in exercising skeletal muscle as well [136, 139]. Also, high-intensity physical exercise brings about cell damage in skeletal muscle, which leads to an infiltration of inflammatory cells such as neutrophils into inflammatory sites in skeletal muscle, resulting in the development of ROS due to NADPH oxidase and myeloperoxidase [135]. Exercise-induced oxidative stress in muscles directly causes muscle damage by oxidation of cell components such as lipids, proteins, and DNA, suggesting to contribute to muscle fatigue, muscle soreness, and exercise-induced muscle injury [140–142]. Many animal studies indicate that ROS is a contributor to contraction-induced muscle fatigue during prolonged endurance exercise, and administration of antioxidants such as NAC is reported to delay muscle fatigue in human subjects [142]. Similarly, several numbers of human studies suggest that supplementation with antioxidant brings about treatment effectiveness to high-intensity exercise-induced muscle damage [143]. In addition, ROS produced during exercise may be involved in delayed-onset muscle damage related to inflammation. For example, oxidative stress and the expressions of proteins for cytokine-induced neutrophil chemoattractant 1 and MCP-1 in rat gastrocnemius significantly increased at 24 h after running exercise, and these increases were significantly ameliorated by supplementation with antioxidant *α*-tocopherol [144]. Meanwhile, it is suggested that increased oxidative stress activates NF-*κ*B, and, as a result, activated NF-*κ*B upregulates the expression of antioxidant enzymes such as Mn-SOD in skeletal muscle [137, 138]. Furthermore, the activity of elevated SOD in skeletal muscle has shown a dependence on the intensity of physical exercise [145–147]. While increases in oxidative stress are observed in not only skeletal muscle but also the liver, heart, and lungs of rats during a maximum single bout of exercise, it is unknown whether oxidative stress in WAT is increased [148]. It is accepted that oxidative stress in the blood increases due to high-intensity physical exercise, and exercise intensity must be relatively high during an examination where the expression of Mn-SOD in WAT increases in animal experiments [111, 112, 132]. Therefore, it is possible that blood oxidative stress leads to an increase in Mn-SOD expression. Other reports, however, have pointed out that when adipocytes are exposed to oxidative stress in vitro, the expression of NADPH oxidase rises; thus further consideration is required [83].

## 8. Conclusions

Reducing oxidative stress in WAT is thought to be essential as a strategy for the prevention and improvement of lifestyle-related diseases, since recent evidence has shown that an increase in oxidative stress is closely related to the dysfunction of WAT due to a dysregulated expression of adipokines. TR is thought to be an important tool for preventing and improving obesity and lifestyle-related diseases, and it is possible that TR attenuates the dysregulated expression of adipokines by reducing the oxidative stress in WAT. However, many points remain unclear and further research is warranted.
